# Up and Down the Hill-Plot: Hydrides of CeGe–Structure and Magnetism

**DOI:** 10.1021/acs.inorgchem.6c02477

**Published:** 2026-08-14

**Authors:** Leonhard Y. Dorsch, Nikolai Haferkorn, Jasper A. Baldauf, Marvin Michak, Thomas C. Hansen, Rainer Pöttgen, Holger Kohlmann

**Affiliations:** † Faculty of Chemistry, Institute of Inorganic Chemistry and Crystallography, Leipzig University, Johannisallee 29, Leipzig 04103, Germany; ‡ Institut für Anorganische und Analytische Chemie, Universität Münster, Corrensstraße 30, Münster 48149, Germany; § Institut Laue Langevin, 71 Avenue des Martyrs, BP 156, Grenoble Cedex 9 F-38042, France

## Abstract

The hydrogenation behavior of FeB type CeGe was investigated. Hydrogenation (deuteration) produces mixtures of two modifications, similar to hydrides of other FeB type phases *LnTt* (*Ln* = La, Ce, Nd; *Tt* = Si, Ge). *P*-CeGeD_0.75(2)_ (*Pnma*, *a* = 8.1603(5) Å, *b* = 4.0938(3) Å, *c* = 6.2911(4) Å) crystallizes in the LaGeH structure type (filled FeB type), whereas *C*-CeGeD_0.95(3)_ (*Cmcm*, *a* = 4.2958(2) Å, *b* = 11.9970(7) Å, *c* = 4.0885(3) Å) crystallizes in the ZrNiH structure type (filled CrB). Similar to CeSiH_*x*_ the mixtures feature exceptionally high ferromagnetic ordering temperatures of 21.6(1) K for CeGeD_*x*_ and 22.1(2) K for CeGeH_*x*_ (*x* ≤ 1), with the effective magnetic moments of 2.44(1) μ_B_ (CeGeD_*x*_) and 2.38(1) μ_B_ (CeGeH_*x*_) being close to the free ion value of 2.54 μ_B_ of Ce^3+^. The hydrides (deuterides) were studied by neutron powder diffraction (NPD), thermal analysis and magnetic susceptibility measurements.

## Graphical abstract

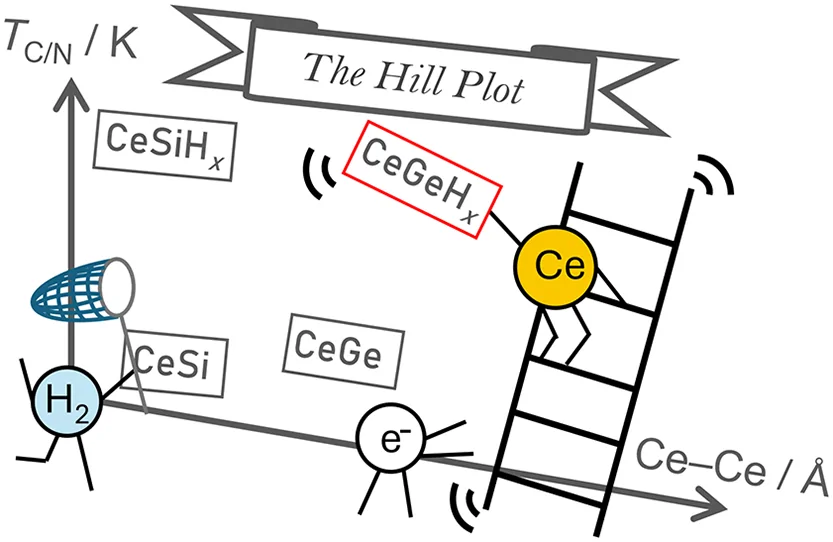

## 1 Introduction

Being “single f” systems, cerium compounds offer a wide range of effects to study, ranging from magnetism to fluctuating valence, heavy Fermions and Kondo effects.^1–6^ A useful tool to visualize the impact of the Ce–Ce distance on the magnetism in intermetallic cerium compounds is the Hill-Plot.^7^ Comparing the shortest Ce–Ce distance in each phase to the ordering temperature, the proposed Hill limit of roughly 3.4 Å can be used to discriminate between superconducting compounds below and magnetic ones above it. The idea was further developed by Koelling^8^ in 1985 and Miyahara et al. in 2018.^9^ The latter study compared more than 700 intermetallic cerium compounds on their distances and magnetic ordering temperatures.^9^ At the time only 14 compounds exhibited ordering temperatures above 18 K.^18^ It was concluded that, in order to reach high ordering temperatures, intermetallic cerium compounds required comparatively short Ce–Ce distances (< 4.6 Å) alongside a suppression of the valence instability in Ce.^9^

An interesting example that follows this rule is the remarkable change in magnetic behavior between CeSi and its hydrides. The FeB type phase CeSi (space group *Pnma*/No. 62)^10–12^ is a commensurate antiferromagnet with a Néel temperature of *T*_N_ = 5.6 K.^13,14^ Upon hydrogenation, it turns into two hydrides with ferromagnetic ordering temperatures of *T*_C_ = 22.4(1) K for the ZrNiH type *C*-CeSiH_*x*_ in space group *Cmcm* and *T*_C_ = 24.2(1) K for the LaGeH type *P*-CeSiH_*x*_ in space group *Pnma.* ^15^ The convention of referring to hydrides in space group *Cmcm* as the *C*-phase or *C*-hydride and *P*-phase or *P*-hydride for hydrides in space group *Pnma* is continued in this work as well. The effective magnetic moment of μ_eff_ = 2.53(1) μ_B_ Ce atom^–1^ indicates a stable trivalent ground state, as the literature value of (μ_eff_(Ce^3+^) = 2.54(1) μ_B_)^16^ is closely matched. While the structural differences appear to be moderate, as the shortest Ce–Ce distance changes from 3.767(2) Å in CeSi to 3.787(3) Å in *P*-CeSiH_*x*_, the electronic structure seems to be the driving force for the drastic change in magnetic properties.^15^

Structurally, the FeB type phases *LnTt* (*Ln* = La, Ce, Nd; *Tt* = Si, Ge) (e.g., Figure 1a) consist of silicon or germanium atoms surrounded by a capped trigonal prism of lanthanoid atoms (Figure 1b,c), arranged in rows of alternating prisms alongside the crystallographic *a* direction to create _∞_^1^[*Tt* ^2–^] zigzag chains (Figure 1d). The excess electron according to a limiting crystal chemical formula *Ln* ^3+^ *Tt* ^2–^e^–^ leads to metallic properties.^19^ These electrons are localized upon hydrogenation, as hydride ions are incorporated in the distorted tetrahedral voids. The hydrogenation can therefore be described as a reduction of free charge carriers, however, even for full hydrogen occupation quantum-mechanical calculations suggest *LnTt*H to be poor metals.^15,17^

**Figure 1:**
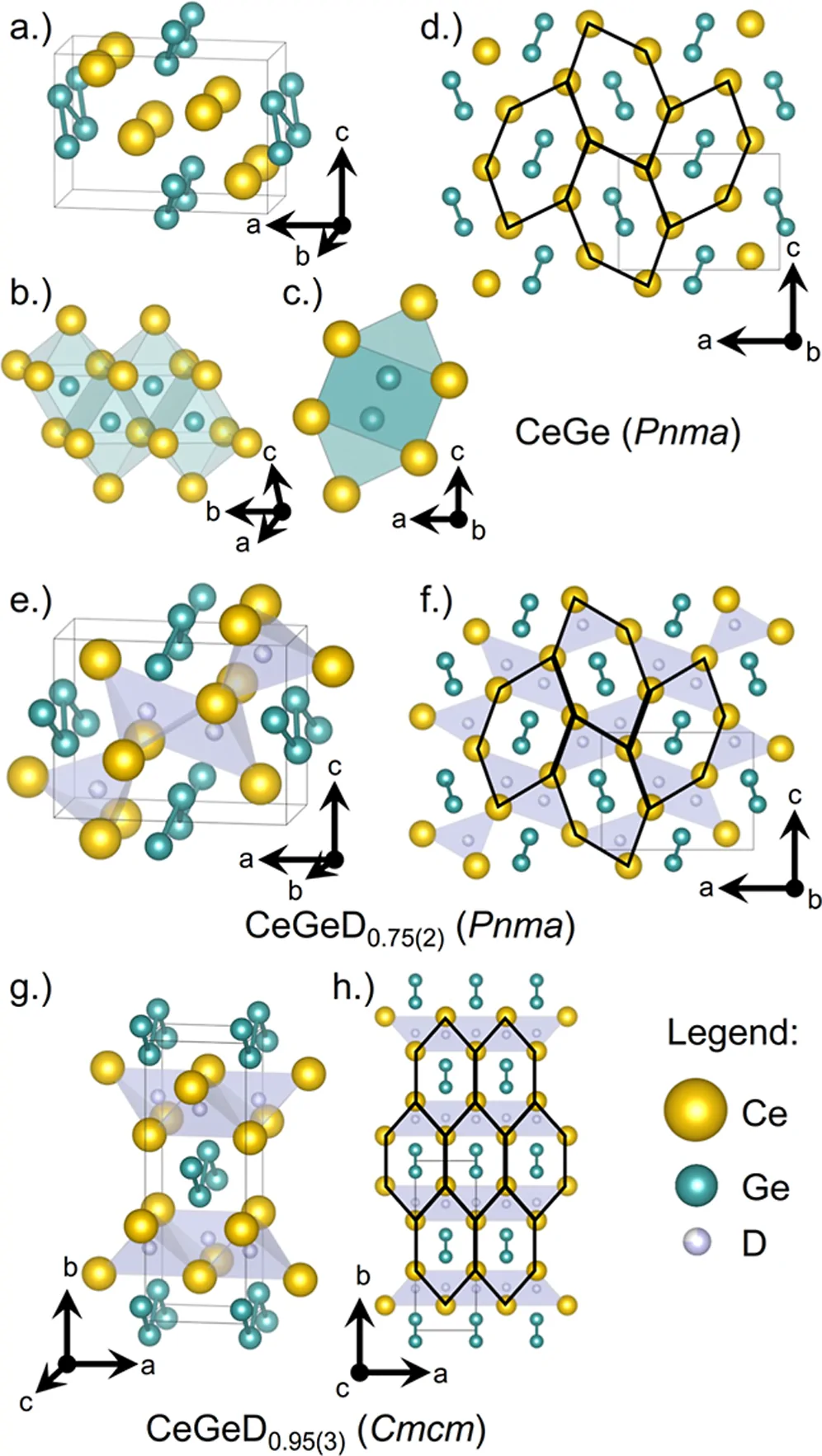
Crystal structures of CeGe (a), CeGeD_0.75(2)_ (e) and CeGeD_0.95(3)_ (g), the capped trigonal prismatic coordination of Ge in CeGe (b,c) and the orientation of alternating GeCe_7_-polyhedra alongside the zigzag chains in CeGe (d), CeGeD_0.75(2)_ (f) and CeGeD_0.95(3)_ (h).

**Table 1:** Crystal Structure Data of CeGe and Selected Interatomic Distances, Determined by XRPD at Room Temperature (Figure S1)

CeGe (*Pnma*): *a* = 8.35398(8) Å, *b* = 4.07949(4) Å, *c* = 6.02681(6) Å, *V* = 205.394(3) Å^3^					
	*x*	*y*	*z*	*B*_iso_/Å_^2^_	sof
Ce	0.1796(2)	1/4	0.1176(2)	0.51(4)	1
Ge	0.0386(2)	1/4	0.6328(5)	0.88(6)	1
Ce–Ce		3.8241(14) Å/ 3.896(2) Å/ 4.07949(4)/4.472(2) Å			
Ge–Ge			2.672(3) Å		
Ge–Ge–Ge			99.53(8)° @2.672(3) Å		

**Figure 2:**
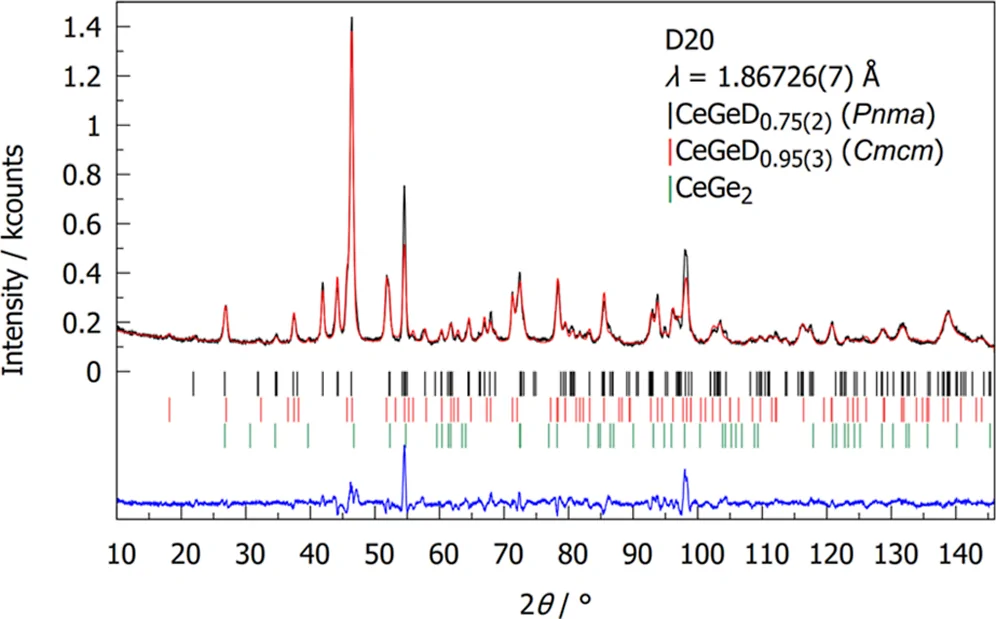
Rietveld refinement of the crystal structures of CeGe deuterides based on neutron powder diffraction data at room temperature, taken at D20 (ILL, Grenoble, France, NUMOR: 283489) at λ = 1.86726(7) Å. *P*-CeGeD_0.75(2)_: 53(2)%, *C*-CeGeD_0.95(3)_: 46(2)%, CeGe_2_ = 0.8(2)%, *R*_p_ = 5.78%, *R*_wp_ = 7.32%, *R*_exp_ = 3.65%, χ^2^ = 4.03.^34^

**Table 2:** Crystal Structure Data of *P*-CeGeD_0.75(2)_ and Selected Interatomic Distances Determined by Neutron Powder Diffraction at Room Temperature (Figure 2)^34^

CeGeD_0.75(2)_ (*Pnma*): *a* = 8.1603(5) Å, *b* = 4.0938(3) Å, *c* = 6.2911(4) Å, *V* = 210.16(2) Å^3^					
	*x*	*y*	*z*	*B*_iso_/Å^2^	sof
Ce	0.178(1)	1/4	0.137(2)	0.8(2)	1
Ge	0.027(1)	1/4	0.612(1)	1.5(2)	1
D	0.377(1)	1/4	0.421(1)	0.3(2)	0.75(2)
Ce–Ce		3.94(1) Å/3.94(1) Å/ 4.0938(3) Å/ 4.32(1) Å			
Ce–D		2.42(1) Å/2.48(1) Å/ 2.497(7) Å			
Ge–Ge		2.524(6) Å			
Ge–Ge–Ge		108.4(2)° @2.524(6) Å			

**Table 3:** Crystal Structure Data of *C*-CeGeD_0.95(3)_ and Selected Interatomic Distances Determined by Neutron Powder Diffraction at Room Temperature (Figure 2)^34^

CeGeD_0.95(3)_ (*Cmcm*): *a* = 4.2958(2) Å, *b* = 11.9970(7) Å, *c* = 4.0885(3) Å, *V* = 210.71(2) Å^3^					
	*x*	*y*	*z*	*B*_iso_/Å^2^	sof
Ce	0	0.3521(6)	1/4	1.6(2)	1
Ge	0	0.0651(4)	1/4	0.9(1)	1
D	0	0.7545(5)	1/4	2.5(2)	0.95(3)
Ce–Ce		3.846(6) Å/ 4.0885(3) Å/ 4.095(9) Å/ 4.2958(2) Å			
Ce–D		2.411(5) Å/2.446(4) Å			
Ge–Ge		2.573(4 ) Å			
Ge–Ge–Ge		105.23(13)° @2.573(4) Å			

**Figure 3:**
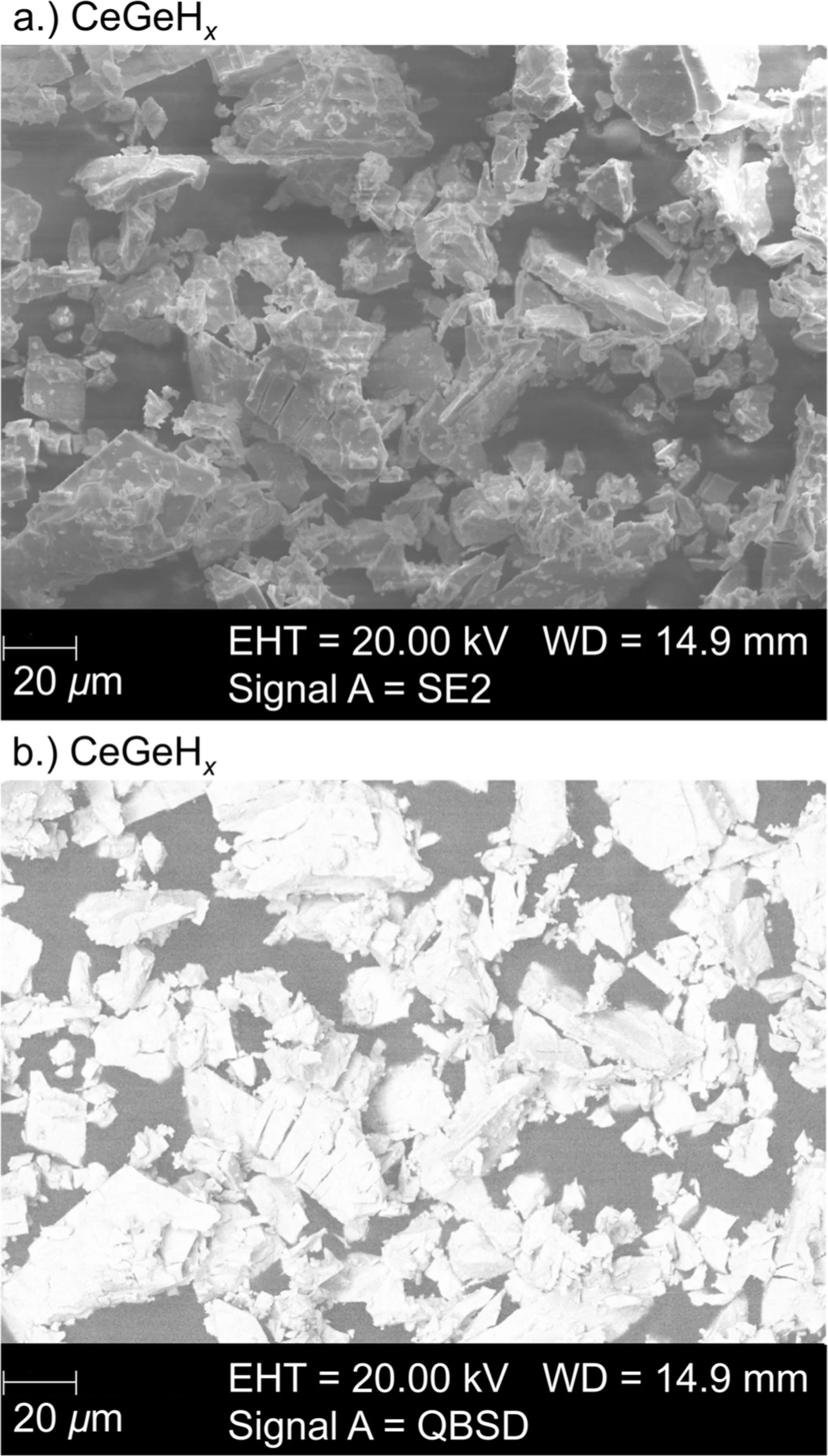
Scanning electron microscope images of CeGeH_*x*_ taken at a scale of 20 μm with a secondary electron detector (a) and a quantum back scatter detector (b).

**Figure 4:**
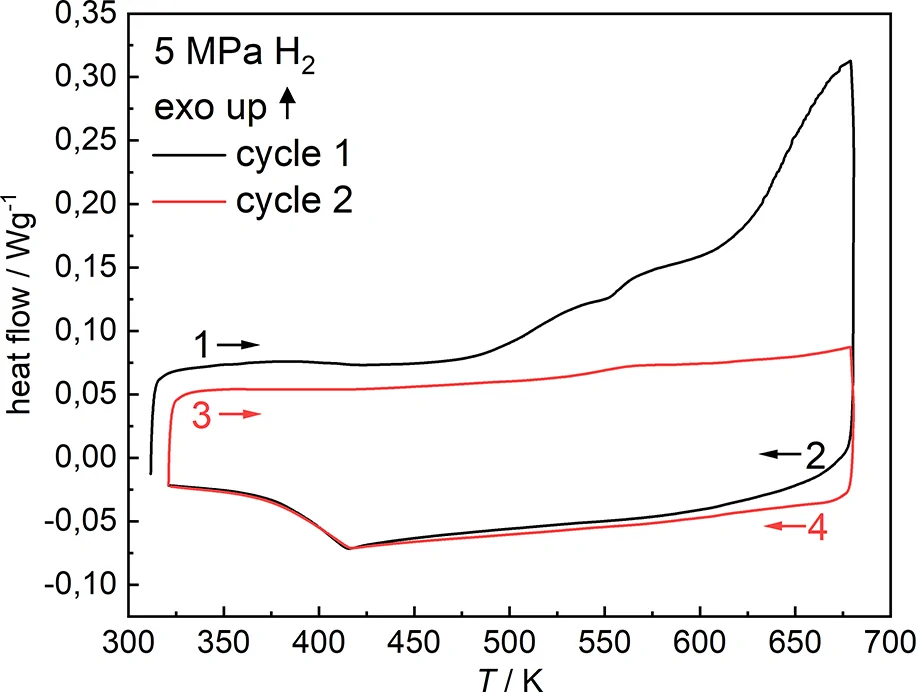
Thermal analysis of the hydrogenation of CeGe at 5 MPa hydrogen pressure, between the first cycle (black) and the second one (red) the temperature of 675 K was maintained for 40 min.

**Figure 5:**
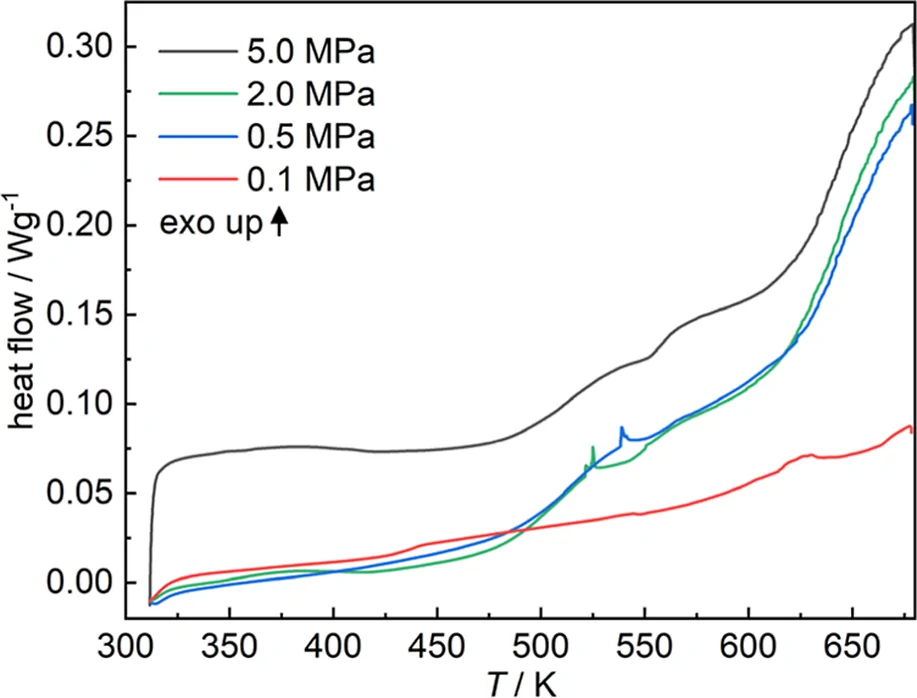
Thermal analysis of the hydrogenation of CeGe at hydrogen pressures between 0.1 and 5 MPa, signals obtained during cooling were omitted for clarity.

The hydrogenation reaction itself unfolds in two stages. In the first step, the overall FeB type structure is retained as the *Ln*_4_ quasi-tetrahedral voids are occupied by hydrogen to form the LaGeH type *P*-hydride in space group *Pnma*. The hydrogenation is accompanied by an increase of the unit-cell volume in the range of 3% with an enlarged *c*, decreased *a* and relatively unchanged *b* axis. Toward higher temperatures, this kinetic product changes into the thermodynamically favored *C*-hydride (ZrNiH type) in space group *Cmcm*, which is comparable to a CrB type structure with filled quasi-tetrahedral voids and slightly larger volumes than the respective *P*-hydrides.^15,17^ Both phases are made up of rows of alternating capped trigonal *Ln* prisms and the _∞_^1^[*Tt* ^2–^] zigzag chains but differ in their arrangement.

As the hydrides of CeSi seem to match the requirements for a high ordering temperature and CeSi as well as LaGe follow the same hydrogenation pathway,^15,17^ CeGe was chosen as an interesting prospect to study and compare the hydrogenation and subsequent magnetic behavior.

Like CeSi, CeGe crystallizes in the orthorhombic FeB structure type (space group *Pnma*/No. 62).^11,20,21^ It exhibits Curie–Weiss paramagnetism, with an antiferromagnetic ordering temperature of *T*_N_ = 10.5 K.^14,22–25^ Polycrystalline CeGe samples can contain tiny amounts of the digermanide CeGe_2_ which is a 7 K antiferromagnet.^26–31^ This is evident in low-field susceptibility measurements as a second anomaly. Single crystal data show a single transition at *T*_N_ and a pronounced metamagnetic transition.^24^

In this work, CeGe was investigated regarding its hydrogenation behavior via differential scanning calorimetry (DSC), neutron powder diffraction (NPD) and scanning electron microscopy (SEM) with energy dispersive X-ray (EDX) spectroscopy. Magnetic characterization was carried out on hydrides and deuterides.

## 2 Experimental Section

### 2.1 Precursor Synthesis

Starting materials for the CeGe synthesis were larger ingots of cerium (Smart Elements, 99.9%) that were cut into pieces and crushed germanium lumps (Haines & Maassen, 99.999%). All elements and samples were stored and handled inside a glovebox under dry argon (Air Liquide, 99.999%) with oxygen and water contents kept below 0.1 ppm. 500 to 1000 mg of equimolar mixtures of Ce and Ge were sealed in welded tantalum jackets. The ampules were then heated in vacuo by induction heating to 1125 K for 300 s, followed by 15 s at 1875 K and a third step at 1475 K for 120 s.

### 2.2 X-ray Powder Diffraction (XRPD)

Samples of CeGe as well as the hydrogenation/deuteration products were fixed in flat sample holders with Lithelen grease between Kapton foils under inert atmosphere and studied on a Guinier geometry G670 camera (HUBER Diffraktionstechnik, Rimsting, Germany) with an image plate detection system. Additional measurements were performed in sealed special glass No. 14 capillaries (Hilgenberg, Malsfeld, Germany) on a STADI P diffractometer (Stoe & Cie, Darmstadt, Germany). Both devices used Cu–K_α1_ radiation.

### 2.3 Hydrogenation with H_2_/D_2_

Hydrogenation reactions were carried out in autoclaves made from Nicofer 5219Nb-alloy containing either H_2_ (Air Liquide, 99.9%) or D_2_ (Air Liquide, 99.8% isotope purity). For the hydrogenation 8 MPa of H_2_ were applied at room temperature and the autoclave was then heated to 575 K for 48 h. The deuteration was carried out with a pressure of 10.8 MPa at room temperature and heated to 575 K for 48 h.

### 2.4 Thermal Analysis

Hydrogenation reactions were also conducted in a TA Instruments Q1000 differential scanning calorimeter (DSC) with a Q10P pressure DSC module. The samples were prepared in crimped aluminum crucibles with 20 mg of sample each. The starting pressures ranged from 0.1 to 5 MPa of H_2_ and temperatures ranged up to 675 K with heating rates of 10 K/min. After each experiment the sample’s phase composition was investigated via XRPD on a Huber G670 diffractometer (*vide supra*). The DSC data was visualized using OriginPro 2019.^32^

### 2.5 Scanning Electron Microscopy

Scanning electron microscopy (SEM) and energy dispersive X-ray (EDX) spectroscopy were performed on CeGe as well as CeGeH_*x*_ samples in a LEO 1530 (Carl Zeiss AG) with a voltage of 20 kV using a liquid nitrogen cooled EDX detector (Oxford Instruments). The ground sample was sputtered with carbon and quickly transferred into the microscope.

### 2.6 Neutron Powder Diffraction

A neutron powder diffraction measurement was carried out at the high-intensity two-axis diffractometer D20 at Institute Laue-Langevin (ILL, Grenoble, France)^33^ with a take-off angle of 118° and equipped with an oscillating collimator. The wavelength was refined with an external Si NIST 640b standard to λ = 1.86726(7) Å. The sample was kept in a thin-walled vanadium cylinder with a 6 mm diameter and indium seals. The data is available under the DOI: 10.5291/ILL-DATA.5–22–844 with the ILL intern raw data labeling NUMOR: 283489.^34^

### 2.7 Rietveld Refinement

The obtained diffraction patterns as well as those measured with XRPD were refined using the FullProf Suite (Vers. 5.10).^35^ The diffraction data was visualized with Gnuplot^36^ while structure figures were created in VESTA.^37^

### 2.8 Magnetic Properties Characterization

Powders of the germanides CeGe, CeGeH_*x*_ and CeGeD_*x*_ are moisture sensitive. They were kept in a glovebox and filled into polypropylene-capsules which were then attached to a brass sample holder rod before being investigated using the VSM (vibrating sample magnetometer) option of a Dynacool Physical Property Measurement System by Quantum Design. The magnetization *M*(*H*, *T*) was measured in the temperature range of 2–305 K using fields up to 90 kOe. Fitting and plotting the data was performed with OriginPro2024 ^38^ and the graphical editing with the program CorelDraw2019.^39^

## 3 Results/Discussion

### 3.1 Structural Analysis

The successful synthesis of CeGe was confirmed by XRPD (Figure S1), with the lattice and structural parameters (Table 1) being close to those reported by Hohnke et al.^12^ It further revealed small impurities of CeGe_2_, which remained unchanged after hydrogenation.

Neutron powder diffraction was performed on a deuterated sample and similarly to other early *LnTt* phases (*Ln* = La, Ce, Nd; *Tt* = Si, Ge)^15,17^ two deuterides in a ratio of roughly 1:1 were found (Figure 2). CeGeD_0.75(2)_ (Figure 1e,f and Table 2) crystallizes in space group *Pnma* in a LaGeH (filled FeB) type structure with the nearly tetrahedral Ce_4_ sites being partially filled by deuterium. Analogous to similar hydrides found for other *LnTt* phases^15,17^ it will be referred to as the *P*-phase or *P*-hydride. It shows a volumetric increase compared to pristine CeGe of 2.3% that is achieved by an anisotropic change in lattice parameters as there is an increase in *c*, a decrease in *a* and a relatively unchanged *b* axis. The second phase CeGeD_0.95(3)_ (Figure 1g,h and Table 3) will be referred to as the *C*-phase or *C*-hydride and crystallizes in space group *Cmcm* in a ZrNiH (filled CrB) type structure with a volumetric increase of 2.6% compared to CeGe. Alongside its higher deuterium content, it shows slightly increased Ge–Ge intrachain distances and decreased angles between the Ge atoms compared to *P*-CeGeD_0.75_. This stands in contrast to the hydrides of CeSi where the Si–Si bonds get shorter and angles wider from the *P*- to the *C*-hydride^15^ but is in line with the comparison between *P*-LaGeD_0.96_ and *C*-LaGeD_0.878_.^17^ As was observed for other deuterides of *LnTt* phases (*Ln* = La, Ce, Nd; *Tt* = Si, Ge),^15,17^ the refinement (Figure 2) showed issues in terms of mismatched intensities. Scanning electron microscopy images (*vide infra*) revealed layered particles that might be the source of the issue due to anisotropic line broadening. A refinement of XRPD data of a hydrogenated sample as well as the corresponding crystal parameters can be found in the Supporting Information (Figure S2; Tables S1, S2)

#### 3.1.1 Scanning Electron Microscopy

Comparing scanning electron microscopic images from CeGe (Figure S3) as well as a hydrogenated sample (Figures 3 and S4) reveals particles broken up in a layered fashion, although not as extensive as previously observed for other filled FeB and CrB type hydrides.^17,40,41^ The mismatched intensities in diffraction patterns (Figure 2) might stem from anisotropic line broadening caused by these layered particles as discussed in refs 40 and 41 or hint at underlying issues with the structural models. This issue also arose for CeSiH_*x*_.^15^ In the backscatter electron SEM image (Figure 3b), the particles show uniform contrast hinting at an even distribution of the elements. The EDX mappings of CeGe and CeGeH_*x*_ (Figures S5 and S6) support a composition of 1:1, the high standard deviations being a consequence of the sample preparation as they were neither pelletized nor polished.

### 3.2 Thermal Analysis

Thermal analysis via DSC at 5 MPa hydrogen gas pressure (Figure 4) revealed exothermic reactions between 475 and 675 K suggesting a hydrogen uptake. After the initial reaction starts, a second step is visible at 550 K, ramping up toward higher temperatures. After 40 min at 675 K neither the cooling phase nor the second cycle showed further signals. The resulting product was confirmed to be a mixture of both CeGeH_*x*_ hydrides via XRPD. The thermal behavior is similar to that of other *LnTt* (*Ln* = La, Ce, Nd, *Tt* = Si, Ge, Sn) phases, as all react exothermically. The two steps reflect the hydrogenation from the FeB type *LnTt* to a filled FeB type *LnTt*H_*x*_ in space group *Pnma* (kinetic product) and a subsequent reaction to a filled CrB type *LnTt*H_*x*_ in space group *Cmcm* (thermodynamic product) at higher temperatures.^15,17^ Respective DSC measurements up to 525 and 585 K followed by XRPD measurements reinforced this picture, as higher *C*-phase contents were observed after higher temperatures. The phase pure synthesis of one of the hydrides was not achieved as the *P*-phase was always accompanied by either CeGe or *C*-CeGeH_*x*_ and sufficient temperatures or reaction times for the *C*-phase were not reached.

To further deepen the understanding of the hydrogenation reaction, the behavior toward lower pressures was studied, as previously only pressures above 2 MPa had been considered.^15,17^ The comparison between thermal analysis measurements at 5.0, 2.0, 0.5, and 0.1 MPa H_2_ pressure (Figure 5) revealed similar hydrogenation pathways down to 0.5 MPa, while the measurement at 0.1 MPa showed little to no signals. Ambient pressures of hydrogen should therefore not suffice for the reaction.

### 3.3 Magnetic Properties

As an additional check we first measured the CeGe sample used for the subsequent hydrogenation and deuteration experiments. The temperature dependence of the inverse magnetic susceptibility is presented in Figure 6a. The experimental magnetic moment of 2.69(1) μ_B_ Ce atom^–1^ is in agreement with the value of 2.54 μ_B_ for the free Ce^3+^ ion^16^ and is also consistent with the single crystal study by Kumar Das et al.^24^ This is also the case for the magnetization behavior (Figure 6b,c). The experimentally derived parameters are summarized in Table 4.

**Figure 6:**
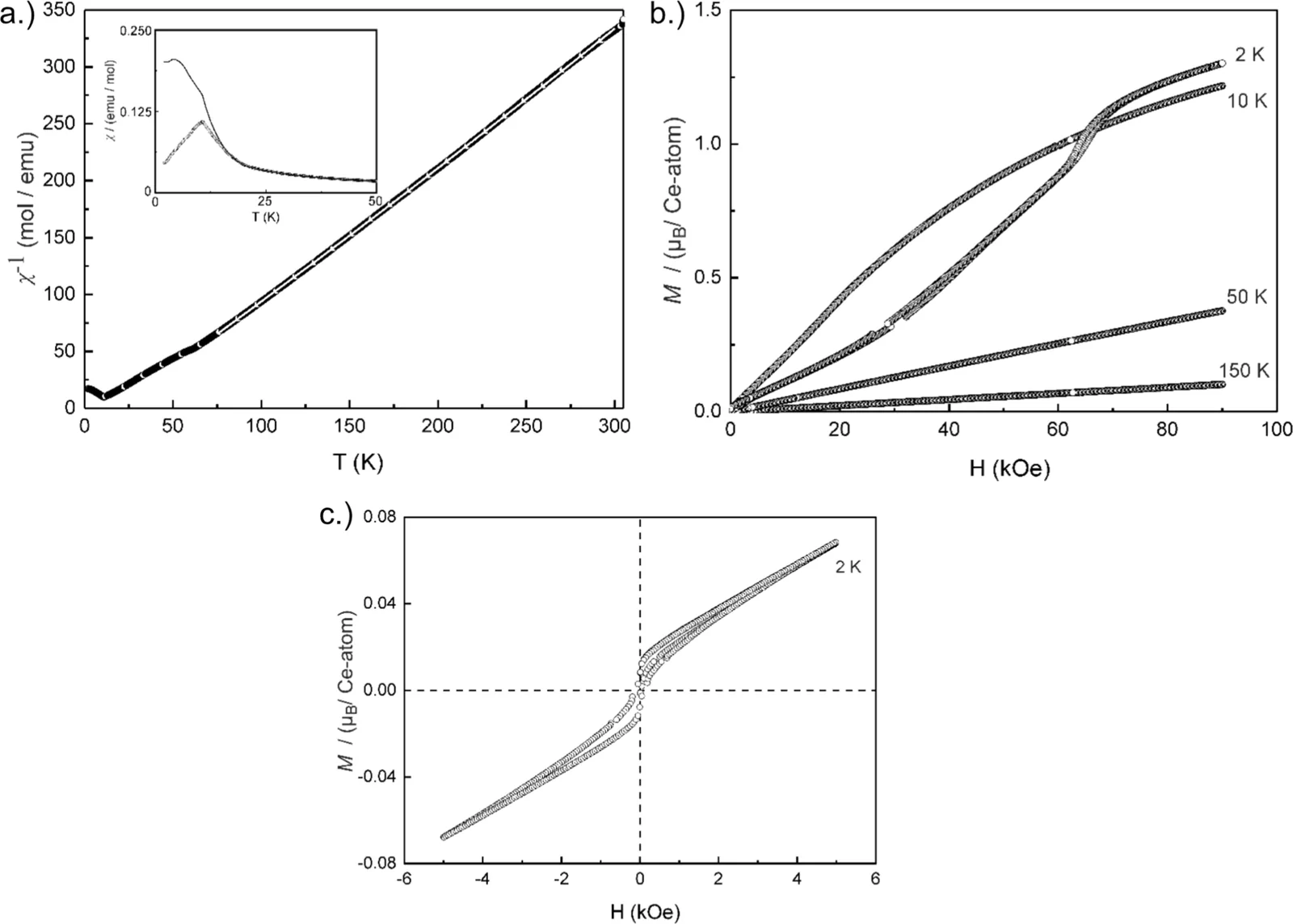
(a) Temperature dependence of the magnetic susceptibility of CeGe measured at 10 kOe zero-field-cooling (ZFC) mode. The temperature range used for the Curie–Weiss fit is emphasized by a thin white line. The inset shows the zero-field-cooling/field-cooling (ZFC/FC) measurement at 100 Oe. (b) Magnetization isotherms of CeGe recorded at 2, 10, 50, and 150 K. (c) Complete hysteresis curve of CeGe at 2 K in the ±5 kOe range.

**Table 4:** Magnetic Properties of CeGe, CeGeH_*x*_ and CeGeD_*x*_: Curie Temperature *T*_C_, Néel Temperature *T*_N_, Effective Magnetic Moment μ_Eff_, Theoretical Magnetic Moment μ_theo_, Paramagnetic Curie Temperature θ_P_, Experimental Saturation Magnetization μ_sat_, Theoretical Saturation Magnetization *g*_J_ × *J*. Data of CeSiH_*x*_ and CeSiD_*x*_ is Taken from ref 15 for Comparison

compound	μ_eff_/μ_B_	μ_theo_/μ_B_	*θ*_P_/K	*T*_C_/K	*T*_N_/K	μ_sat_/μ_B_	*g*_*J*_ × *J*
CeGe	2.69(1)	2.54	16.7(1)	–	10.8(1)	1.30(1)	2.14
CeGeH_*x*_	2.38(1)	2.54	17.9(1)	22.1(2)		1.08(1)	2.14
CeGeD_*x*_	2.44(1)	2.54	19.9(1)	21.6(1)		1.19(1)	2.14
CeSiH_*x*_	2.53(1)	2.54	25.3(1)	24.3(1)		1.32(1)	2.14
CeSiD_*x*_	2.40(2)	2.54	55.8(1)	24.7(1)		1.40(1)	2.14

We now switch to the magnetic properties of the hydrogenated CeGeH_*x*_ sample. The temperature dependence of the inverse susceptibility and the low-temperature behavior are shown in Figure 7a and its inset. CeGeH_*x*_ keeps the stable trivalent ground state. This is evident from the experimental magnetic moment of 2.38(1) μ_B_ Ce atom^–1^. The striking feature is the switch of magnetic ordering to a ferromagnetic state as a consequence of the hydrogenation reaction. The low-field measurement in zero-field-cooling/field-cooling (ZFC/FC) mode at 100 Oe revealed a Curie temperature of *T*_C_ = 22.1(2) K. The magnetization behavior of CeGeH_*x*_ is shown in Figure 7b. At 50 and 150 K, well above the Curie temperature, we observe a monotonous increase of the magnetization with low absolute value, characteristic for a paramagnetic material. In the ferromagnetically ordered state (2 and 10 K isotherms), the magnetization shows a steep increase already at low external field strengths and a rapid trend to saturation. This is characteristic for a soft ferromagnet. At 2 K and 90 kOe, the highest magnetization value of 1.08(1) μ_B_ Ce atom^–1^ is still lower than the maximal possible saturation magnetization of 2.14 μ_B_ Ce atom^–1^ according to *g*_J_ × *J*.^16^ The moment reduction is a consequence of crystal field splitting of the *J* = 5/2 ground state. Such reduced saturation magnetizations have been observed in almost all equiatomic Ce*TX* compounds (*T* = transition metal; X = *p* element of the third, fourth or fifth main group).^42–44^

**Figure 7:**
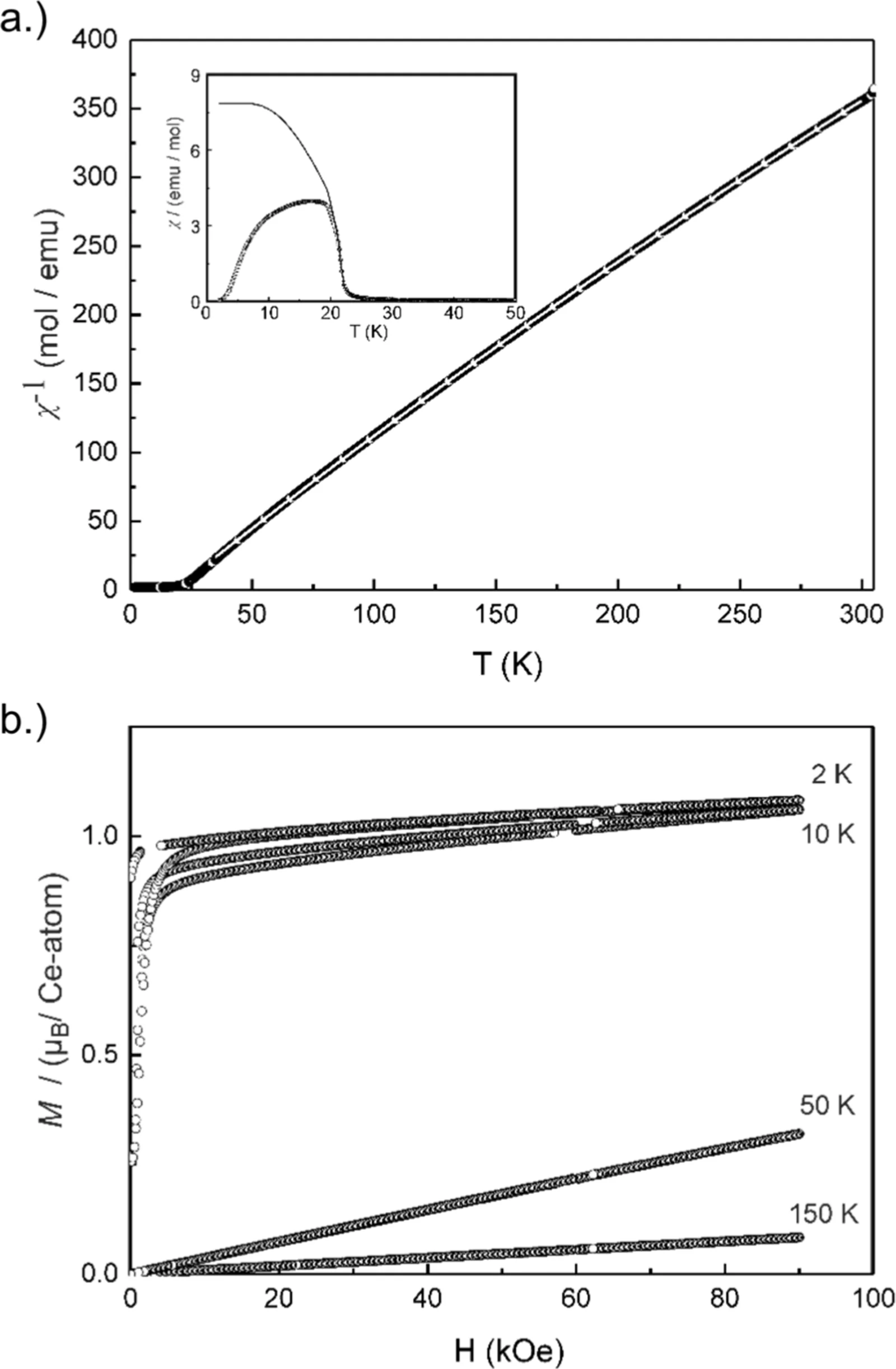
(a) Temperature dependence of the inverse magnetic susceptibility of the CeGeH_*x*_ sample (10 kOe data). The temperature range used for the Curie–Weiss fit is emphasized by a thin white line. The inset shows the zero-field-cooling/field-cooling (ZFC/FC) measurement at 100 Oe. (b) Magnetization isotherms of CeGeH_*x*_ recorded at 2, 10, 50, and 150 K.

The CeGeD_*x*_ sample shows comparable behavior (Figure 8; Table 4). The Curie temperature of *T*_C_ = 21.6(1) K is only slightly lower than the one observed for the hydrogenated sample. The magnetic behavior of CeGeH_*x*_ and CeGeD_*x*_ is similar to CeSiH_*x*_ and CeSiD_*x*_,^15^ however, with slightly lower Curie temperatures (Table 4). The remarkable feature of these four cerium-based tetrelides is their comparatively high magnetic ordering temperature. Only few other intermetallics show higher ones (Table 5).

**Figure 8:**
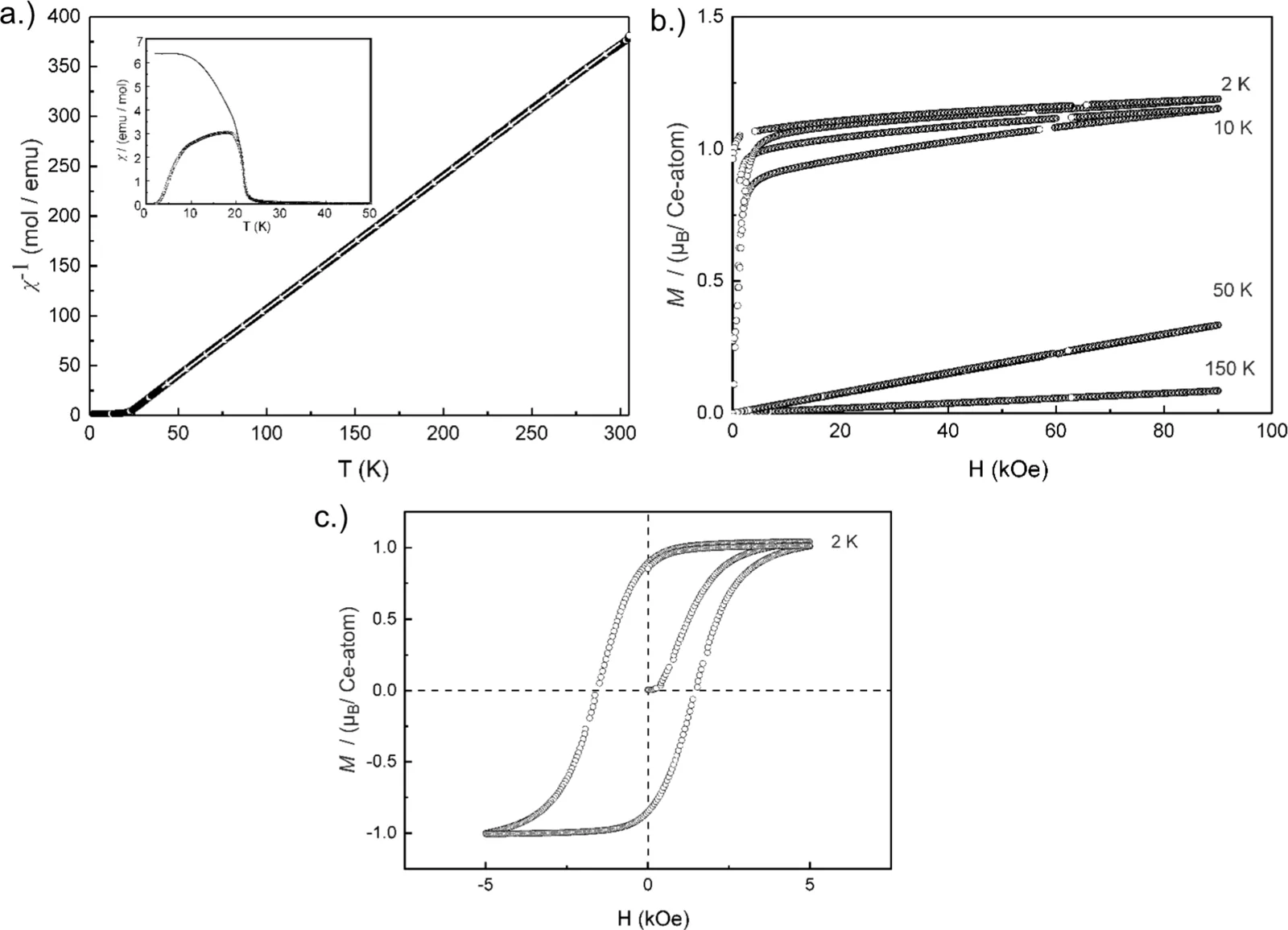
(a) Temperature dependence of the inverse magnetic susceptibility of the CeGeD_*x*_ sample (10 kOe data). The temperature range used for the Curie–Weiss fit is emphasized by a thin white line. The inset shows the zero-field-cooling/field-cooling (ZFC/FC) measurement at 100 Oe. (b) Magnetization isotherms of CeGeD_*x*_ recorded at 2, 10, 50, and 150 K. (c) Complete hysteresis curve of CeGeD_*x*_ at 2 K in the ±5 kOe range.

**Table 5:** Magnetic Ordering Temperatures (*T*_m_) and Magnetic Ground State (AF, Antiferromagnetism; F, Ferromagnetism) of the Highest Ordering Cerium Intermetallics (Extended Version of Table 1 from refs 9)

compound	ground state	Tm/*K*	compound	ground state	Tm/*K*
Ce_5_NiPb_3_ ^45^	F	48	CeBi^46^	AF	26
Ce_5_Pb_3_O^47^	F	46	CeScSi^48^	AF	26
Ce_5_CuPb_3_ ^49^	F	46	CeSiH_*x*_ ^15^	F	24.3(1)
CeScGe^48^	AF	46	CeSiD_*x*_ ^15^	F	24.7(1)
Ce_2_AuP_3_ ^9,50^	F	31	CeGeH_*x*_	F	22.1(2)
CeZn^51^	AF	30	CeGeD_*x*_	F	21.6(1)
CeC_2_ ^52^	AF	30	CeCoGe_3_ ^53,54^	F	21

In 1970 Hill proposed a distance criterion relying on the shortest Ce–Ce spacing in a given crystal structure (the so-called *Hill limit*)^7^ for discriminating between superconductivity and magnetic ordering in cerium intermetallics. At ca. 3.4 Å Ce–Ce spacing the *Hill limit* discriminates the superconducting phases below and the magnetically ordering ones above. Further systemization of the magnetic properties of cerium intermetallics by Koelling^8^ and in a broader context by Miyahara et al.^9^ revealed that only few intermetallic cerium compounds exhibit magnetic ordering above 20 K. The prerequisites for a high Curie temperature for an intermetallic cerium compound are shorter Ce–Ce distances along with a suppression of a valence instability. The recently reported silicide hydride CeSiH_*x*_ ^15^ and the germanide hydride CeGeH_*x*_ fit in this series.

Given that CeSi and CeGe form two hydrides each and the respective deuterides are easily available as well, a Hill-Plot was created to see if trends emerge within these compounds (Figure 9, Table 6). While the step from the antiferromagnetic intermetallic phases to the ferromagnetic hydrides is quite pronounced, as the hydrogenation suppresses valence instability in Ce, a differentiation within the hydrides remains difficult. As shorter Ce–Ce distances are correlated to higher ordering temperatures, substituting Si for the larger Ge should increase the Ce–Ce distances and thus lower *T*_C_. Indeed, the silicides generally show smaller Ce–Ce distances and higher ordering temperatures than the germanides. In the same way one would expect the deuterides to exhibit ordering at higher temperatures than the hydrides, due to a reduced thermal vibration of the heavier isotope. This is, however, only observed for the silicides as within the germanides the hydrides exhibit higher Curie temperatures. The underlying reason might be slightly differing hydrogen (deuterium) contents.

**Figure 9:**
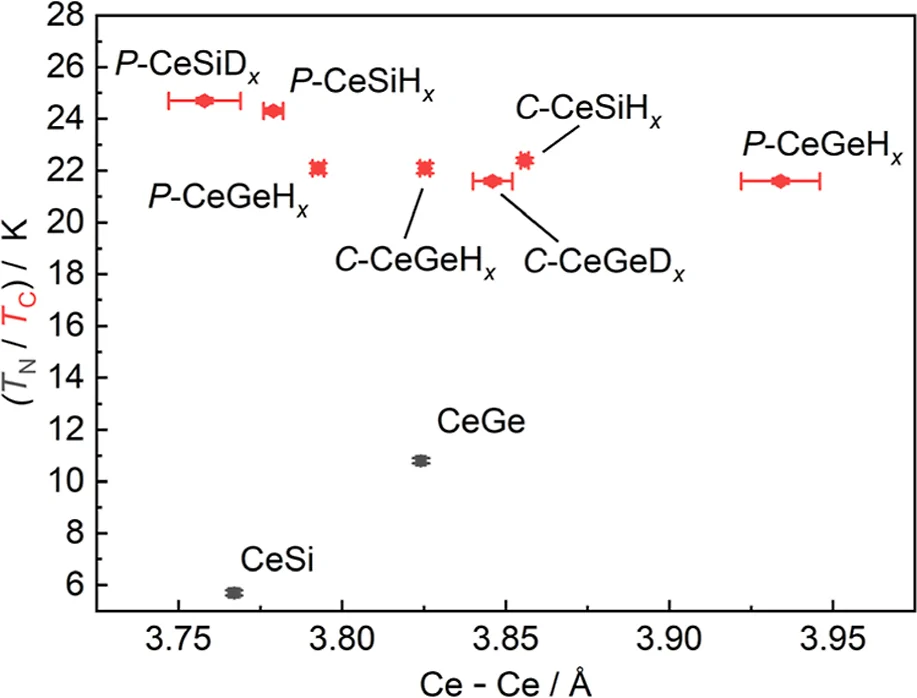
Hill-Plot of CeSi, CeGe and their hydrides and deuterides. Values for CeSi and the respective hydrides were taken from^15^ *C*-CeSiD_*x*_ was omitted as the ZFC/FZ curves did not allow the accurate determination of *T*_C_.

**Table 6:** Shortest Ce–Ce Distances and Critical Temperatures of CeSi, CeGe and Their Hydrides at Room Temperature^a^

compound	Ce–Ce distance (RT)/Å	*T*_C_/*K*	*T*_N_/*K*
CeSi^15^	3.767(2)		5.7(1)
*P*-CeSiD_*x*_ ^15^	3.758(11)	24.7(1)	
C-CeSiD_*x*_ ^15^	3.902(3)		
*P*-CeSiH_*x*_ ^15^	3.779(3)	24.3(1)	
C-CeSiH_*x*_ ^15^	3.8558(12)	22.4(1)	
CeGe	3.824(1)		10.8(1)
*P*-CeGeD_*x*_	3.94(1)	21.6(1)	
C-CeGeD_*x*_	3.846(6)	21.6(1)	
*P*-CeGeH_*x*_	3.793(2)	22.1(2)	
C-CeGeH_*x*_	3.825(2)	22.1(2)	

a Values for CeSi and the respective hydrides were taken from.^15^ *C*-CeSiD_*x*_ was omitted as the ZFC/FZ Curves did not allow the accurate determination of *T*_C_. The corresponding hill-plot can be seen in (Figure 9)

The Hill-Plot in this form should be interpreted with caution as hydrogen contents can vary over time, altering not only the physical but also the electronic structure. As the differences between phases are quite small an accurate plot would require studies about the shelf life of the hydrides and potentially the crystallographic as well as magnetic characterization to be carried out within a short time frame. Further corrections to account for coordination numbers as shown by Koelling^8^ or the anisotropy of the lattice expansion were also not applied. It also relies on the assumption that the magnetism of the *P*- and *C*-germanide hydride is in fact analogous to the corresponding silicide hydride. This would warrant low temperature NPD measurements, preferably with single-phase samples. Apart from experimental studies, gaining insights about the magnetic ground states through theoretical calculations would also allow to further deepen the understanding of the influence of hydrogenation on the magnetism.

## 4 Conclusion

The hydrogenation of FeB type CeGe can be achieved by applying at least 0.5 MPa hydrogen pressure and temperatures above 475 K. Thermal analysis shows an exothermic two step reaction, first into an LaGeH type *P*-hydride in space group *Pnma* (CeGeD_0.75(2)_: *a* = 8.1604(5) Å, *b* = 4.0938(3) Å, *c* = 6.2910(4) Å) and second in a ZrNiH type *C*-hydride in space group *Cmcm* (CeGeD_0.95(3)_: *a* = 4.2958(2) Å, *b* = 11.9966(7) Å, *c* = 4.0886(3) Å). They resemble variants of the FeB and CrB type, both with hydrogen-filled Ce_4_ quasi-tetrahedral sites. Their volumetric increases as well as the anisotropic expansion of lattice parameters between CeGe and *P*-CeGeH_*x*_, and the tendency for the *C*-hydride to retain higher hydrogen contents are analogous to previously described hydrogenation products of FeB type *LnTt* (*Ln* = La, Ce, Nd; *Tt* = Si, Ge) phases. They also share the same morphology of brittle layers, possibly causing intensity issues in the powder diffraction patterns.^15,17^ A phase pure synthesis of either of the two phases was not achieved, however, given the similarities to the hydrides of CeSi it can be assumed that *C*-CeGeH_*x*_ is the thermodynamically favored modification.^15^

The similarities to CeSiH_*x*_ also extend into the magnetism, as the antiferromagnetic CeGe with a Néel temperature of *T*_N_ = 10.8(1) K switches to ferromagnets with an exceptionally high Curie temperature of *T*_C_ = 22.1(2) K upon hydrogenation. The Curie temperatures of *C*-CeGeH_*x*_ and *P*-CeGeH_*x*_ do not seem to differ much between the modifications, both exhibit an effective magnetic moment close to the free Ce^3+^ ion value of 2.54 μ_B_. The magnetic structures are yet to be determined, however analogous structures to CeSiH_*x*_ with an orientation of the magnetic moments parallel to the Ge–Ge zigzag chains would not be surprising. The reason for the change in magnetic behavior cannot be explained by the changes in lattice parameters and Ce–Ce distances alone. For CeSiH_*x*_, we proposed that they are predominantly caused by the localization of the Ce-d electrons by hydrogen, which suppresses the Kondo screening of 4f electrons and thus enables stronger exchange interactions at higher temperatures.^15^ As the magnetic behavior is generally consistent with CeSiH_*x*_, the same can be proposed for CeGeH_*x*_.

Together with the recently reported CeSiH_*x*_, CeGeH_*x*_ is therefore a prime example of how hydrogenation reactions not only influence crystal structures but can also drastically impact physical properties through changes in the electronic structure.

## Supplementary Material



## Notes

Rietveld analysis on the XRPD powder diffraction patterns of CeGe and CeGeH_*x*_, structural parameters of *P*- and *C*-CeGeH_*x*_, SEM and EDX data of CeGe and CeGeH_*x*_ (PDF)
