# Supplementary material for: Up and Down the Hill-Plot: Hydrides of CeGe–Structure and Magnetism
Source: Inorg Chem. 2026 Aug 14;65(34):19821–9. doi: 10.1021/acs.inorgchem.6c02477 (PMC13536884; doi:10.1021/acs.inorgchem.6c02477)
Supplement: Supplementary file 1 [file ic6c02477_si_001.pdf]

Supporting Information: Up and down the Hill-Plot: Hydrides of CeGe – Structure and magnetism

Leonhard Y. Dorsch,[a] Nikolai Haferkorn,[a] Jasper A. Baldauf,[b] Marvin Michak,[a] Thomas C. Hansen,[c] Rainer Pöttgen,[b] Holger Kohlmann[*a]*

*Corresponding Author

a Leipzig University, Faculty of Chemistry, Institute of Inorganic Chemistry and Crystallography, Johannisallee 29, 04103 Leipzig, Germany. E-mail: holger.kohlmann@uni-leipzig.de

b Institut für Anorganische und Analytische Chemie, Universität Münster, Corrensstraße 30, 48149 Münster, Germany

c Institut Laue Langevin, 71 Avenue des Martyrs, BP 156, F-38042 Grenoble Cedex 9, France

Supporting Information description

Rietveld analysis on the X-ray powder diffraction patterns of CeGe and $CeGeH_x$, structural parameters of *P*- and *C*-$CeGeH_x$, SEM and EDX data of CeGe and $CeGeH_x$ (PDF).

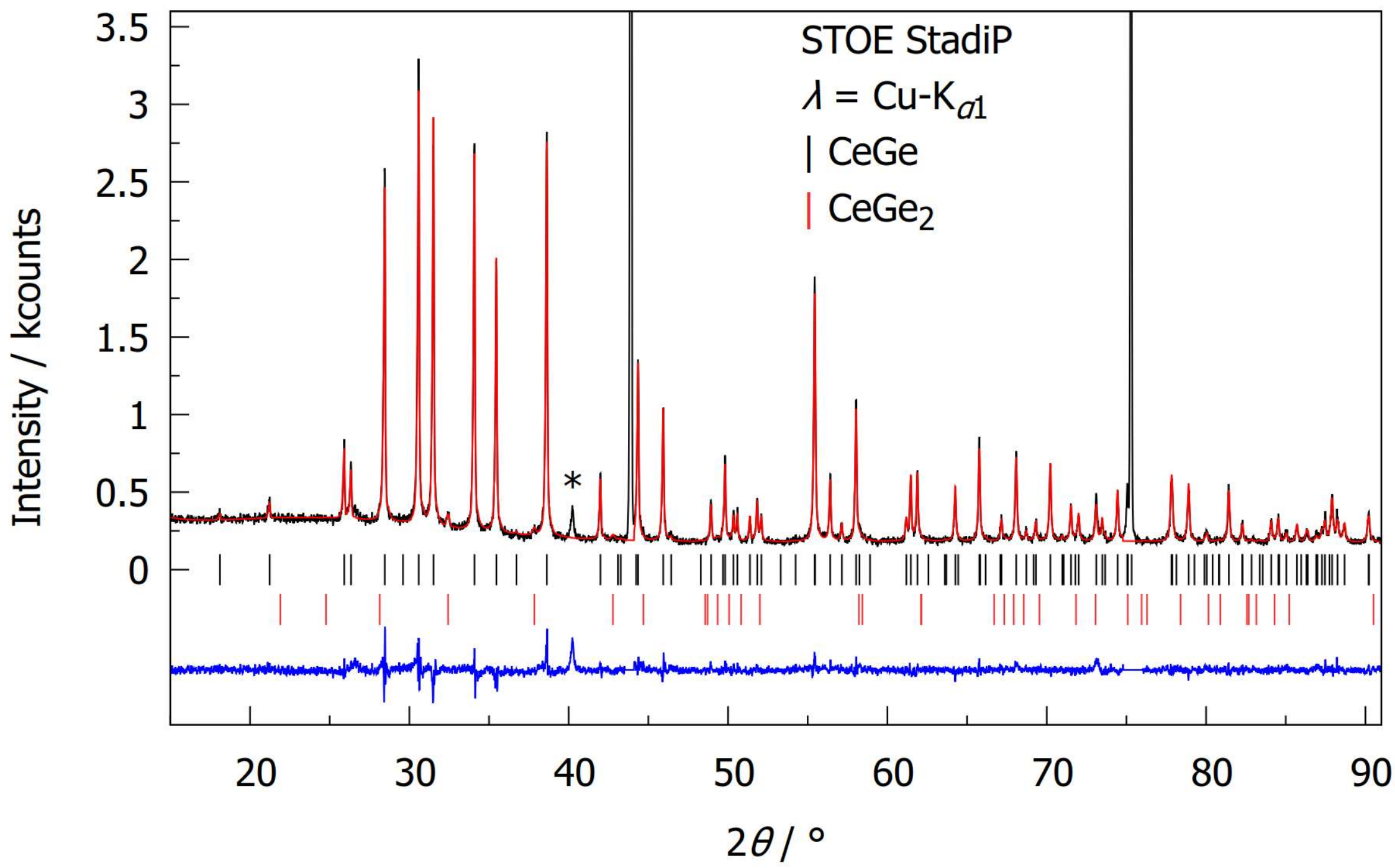

Figure S1. Rietveld refinement of the CeGe crystal structure on an X-ray powder diffraction pattern, Stoe StadiP, reflections of diamond were omitted. CeGe: 98.5(7) w.-%, $CeGe_2$: 1.5(1) w.-%, R_p = 5.15 %, R_{wp} = 6.82 %, χ^2 = 1.37, * marks a parasitic reflection stemming from the sample environment.

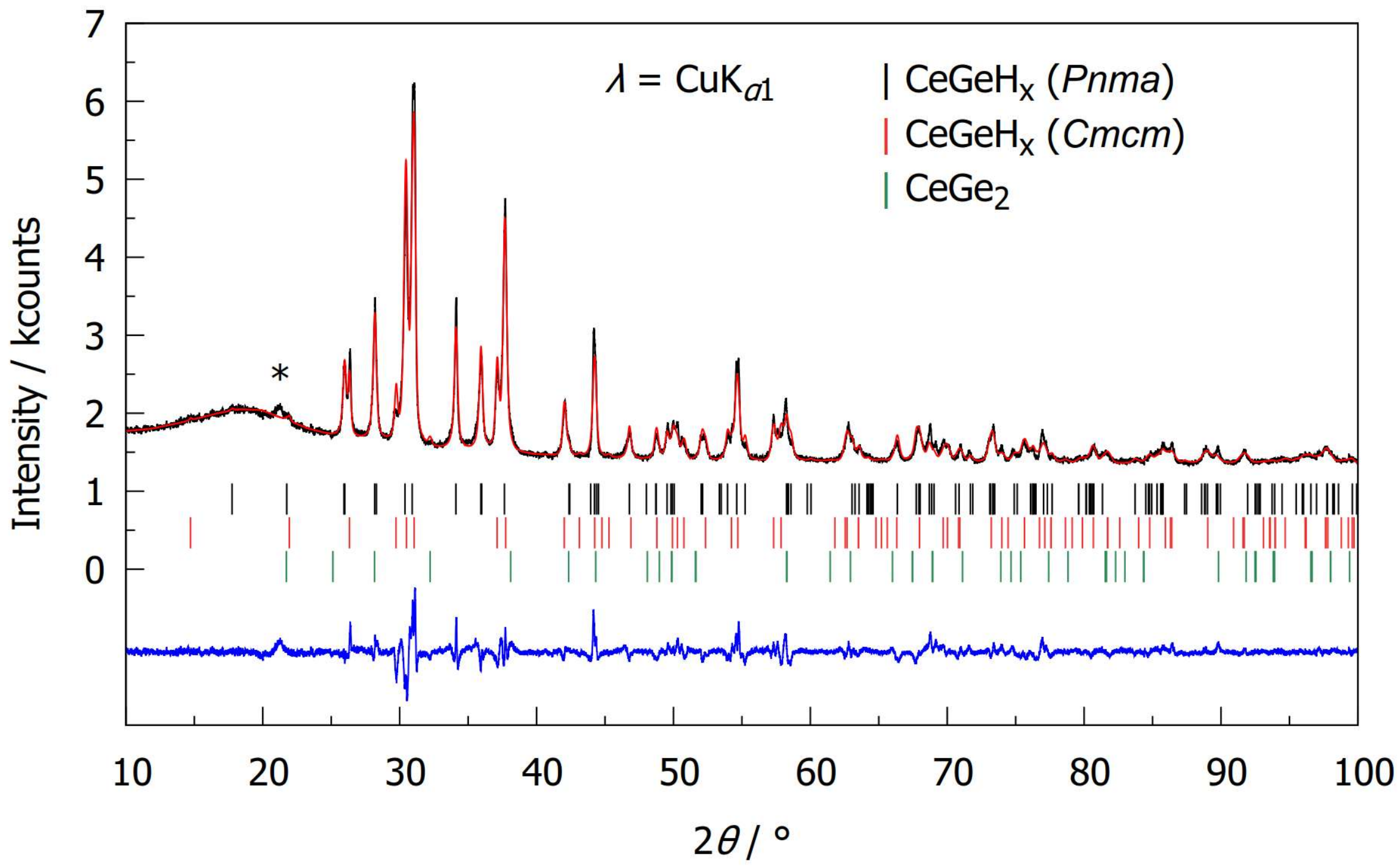

Figure S2. Rietveld refinement of the $CeGeH_x$ crystal structures based on a X-ray powder diffraction pattern, Huber G670, *P*-$CeGeH_x$: 60.4(9) w.-%, *C*-$CeGeH_x$: 38.8(6) w.-%, $CeGe_2$: 0.80(6) w.-%, R_p = 2.21 %, R_{wp} = 3.28 %, χ^2 = 1.81, * marks a parasitic reflection caused by Lithilen® grease.

Table S1. Crystal structure data of *P*-$CeGeH_x$ and selected interatomic distances determined by X-ray powder diffraction at room temperature (Figure S2). Values for hydrogen atoms were taken from the refinement of *P*-$CeGeD_{0.75(2)}$ on neutron diffraction data (Figure 2).

$CeGeH_x$ (*Pnma*)

a = 8.1523(4) Å, b = 4.0895(2) Å, c = 6.2939(3) Å, V = 209.83(2) Å^3

	x	*y*	*z*	B_{iso} / Å^2	*sof*
Ce	0.1715(2)	¼	0.1227(2)	0.43(9)	1
Ge	0.0293(4)	¼	0.6274(7)	1.7(1)	1
H	0.3771	¼	0.4210	1	0.75
Ce–Ce			3.793(2) Å / 3.965(2) Å / 4.0895(2) Å / 4.380(2) Å		
Ge–Ge					2.642(4) Å
Ge–Ge–Ge					101.4(1) ° @ 2.642(4) Å

Table S2. Crystal structure data of *C*-$CeGeH_x$ and selected interatomic distances determined by X-ray powder diffraction at room temperature (Figure S2). Values for hydrogen atoms were taken from the refinement of *C*-$CeGeD_{0.95(3)}$ on neutron diffraction data (Figure 2).

$CeGeH_x$ (*Cmcm*)

a = 4.2929(2) Å, b = 11.9949(6) Å, c = 4.0875(2) Å, V = 210.48(2) Å^3

	x	*y*	*z*	B_{iso} / Å^2	*sof*
Ce	0	0.3508(1)	¼	0.4(1)	1
Ge	0	0.0659(2)	¼	0.5(2)	1
H	0	0.7545	¼	1	0.95
Ce–Ce			3.825(2) Å / 4.0875(3) Å / 4.121(2) Å / 4.2929(2) Å		
Ge–Ge					2.584(2) Å
Ge–Ge–Ge					104.55(7) ° @ 2.584(2) Å

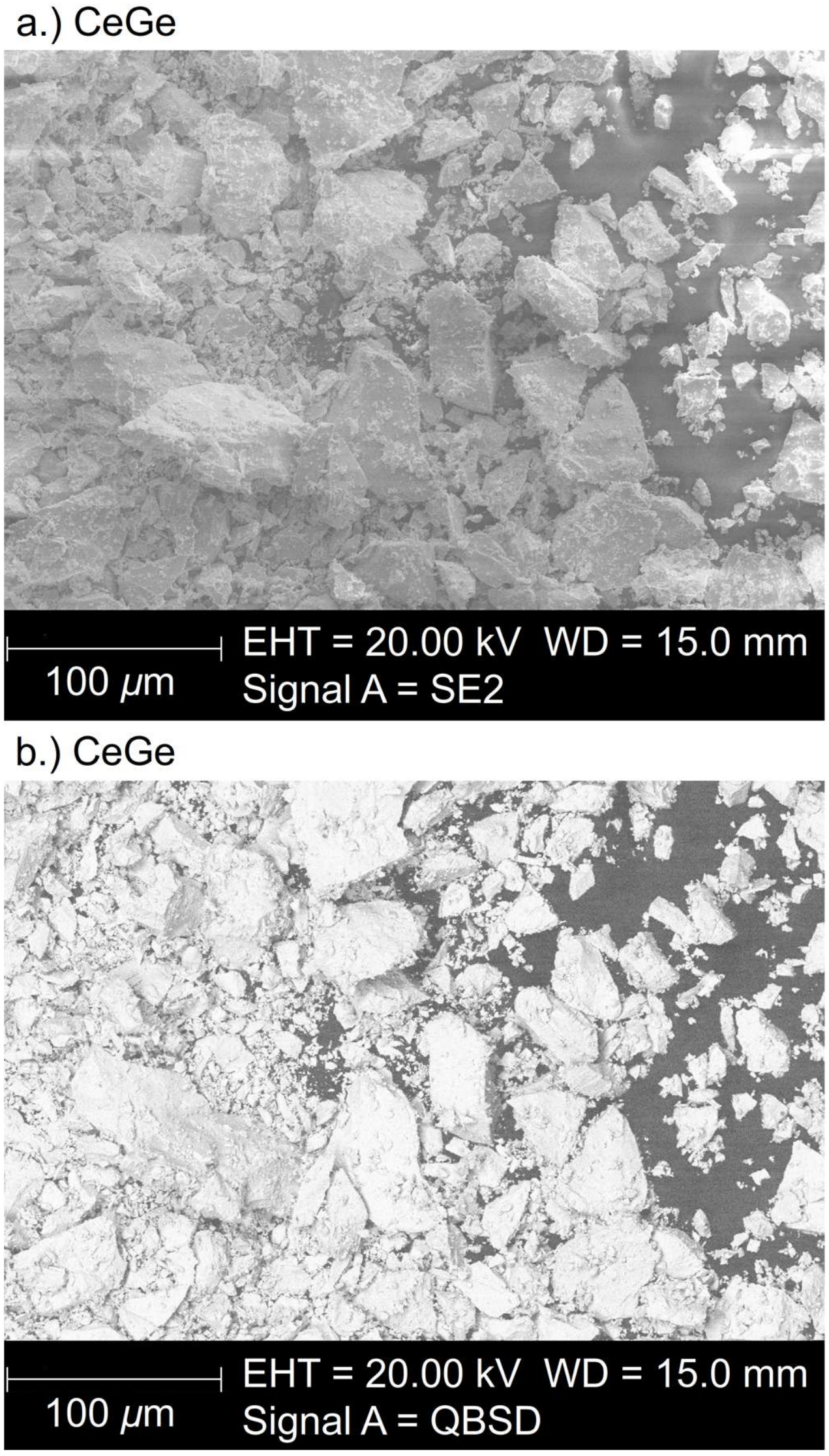

Figure S3. Scanning electron microscope images of CeGe taken at a scale of 100 μm with a secondary electron detector (a) and a quantum back scatter detector (b).

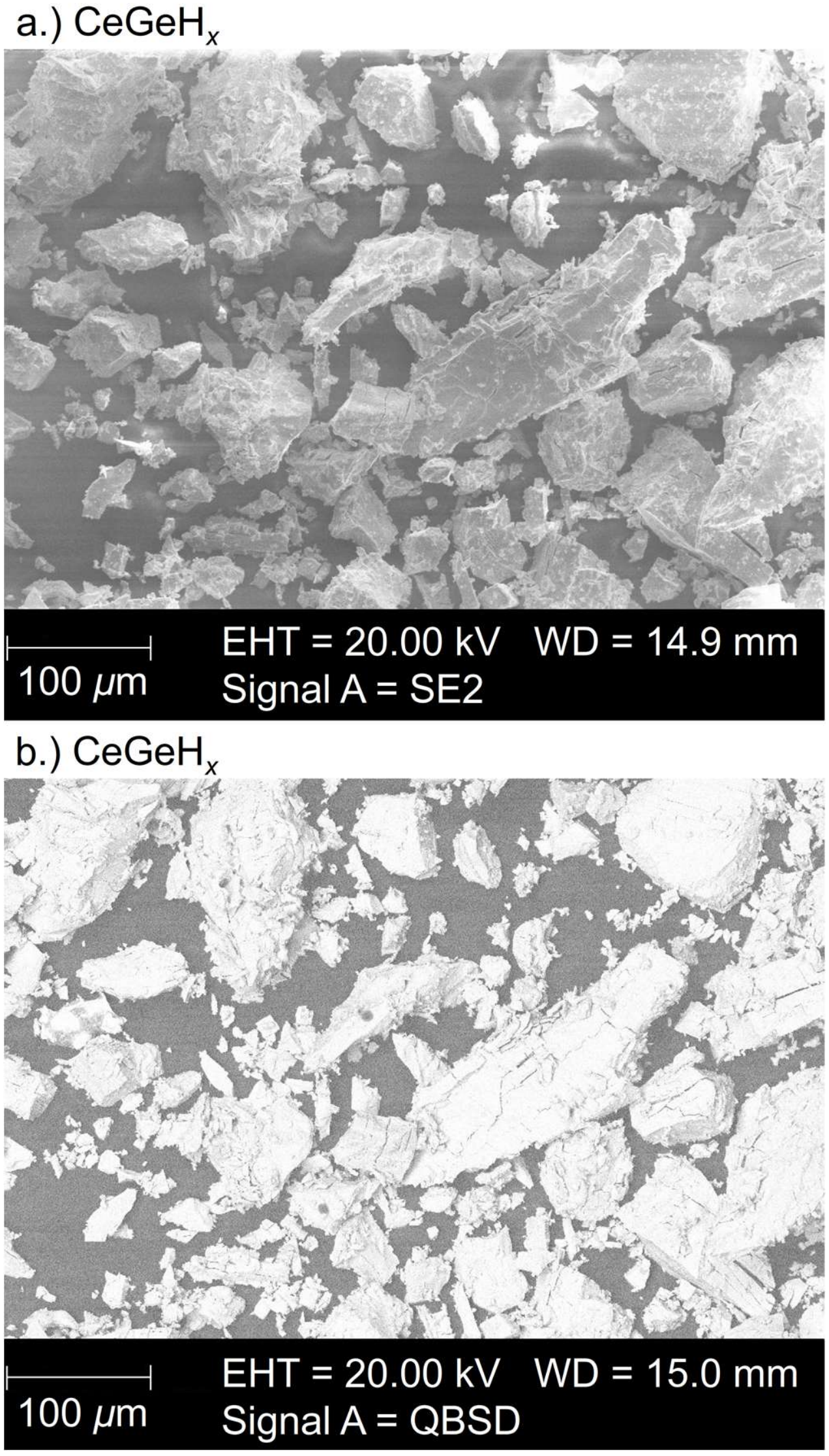

Figure S4. Scanning electron microscope images of $CeGeH_x$ taken at a scale of 100 μm with a secondary electron detector (a) and a quantum back scatter detector (b).

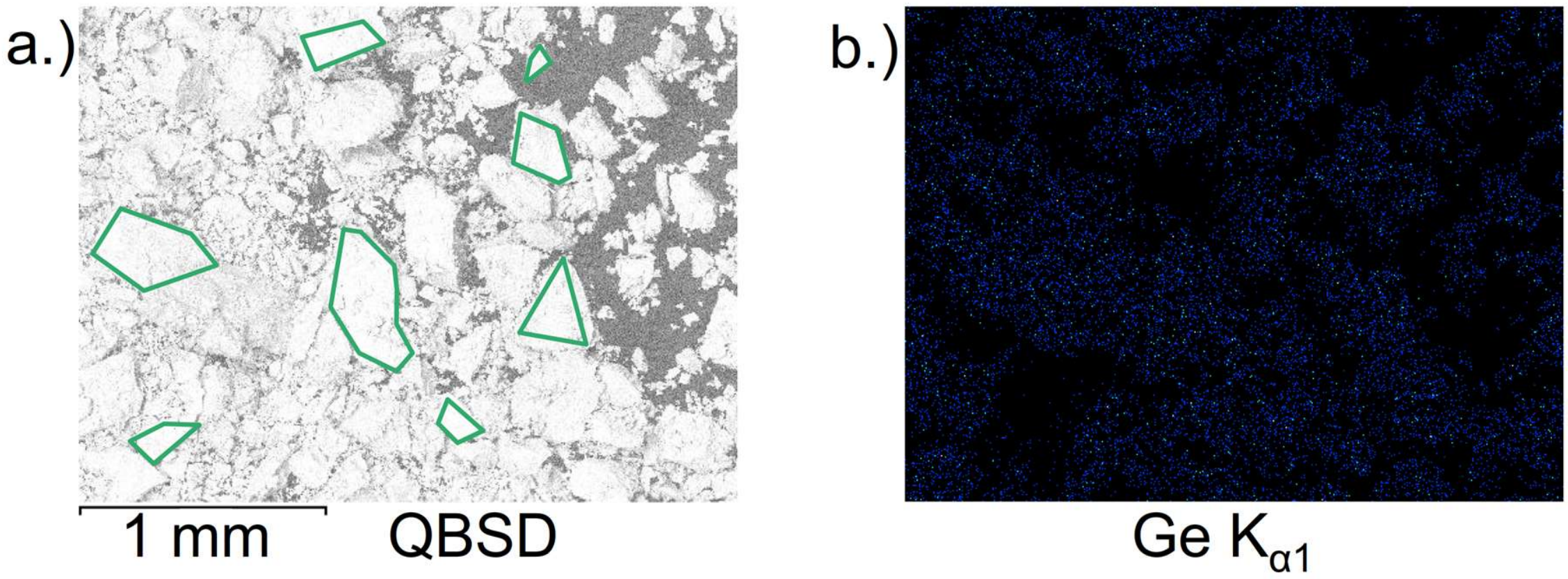

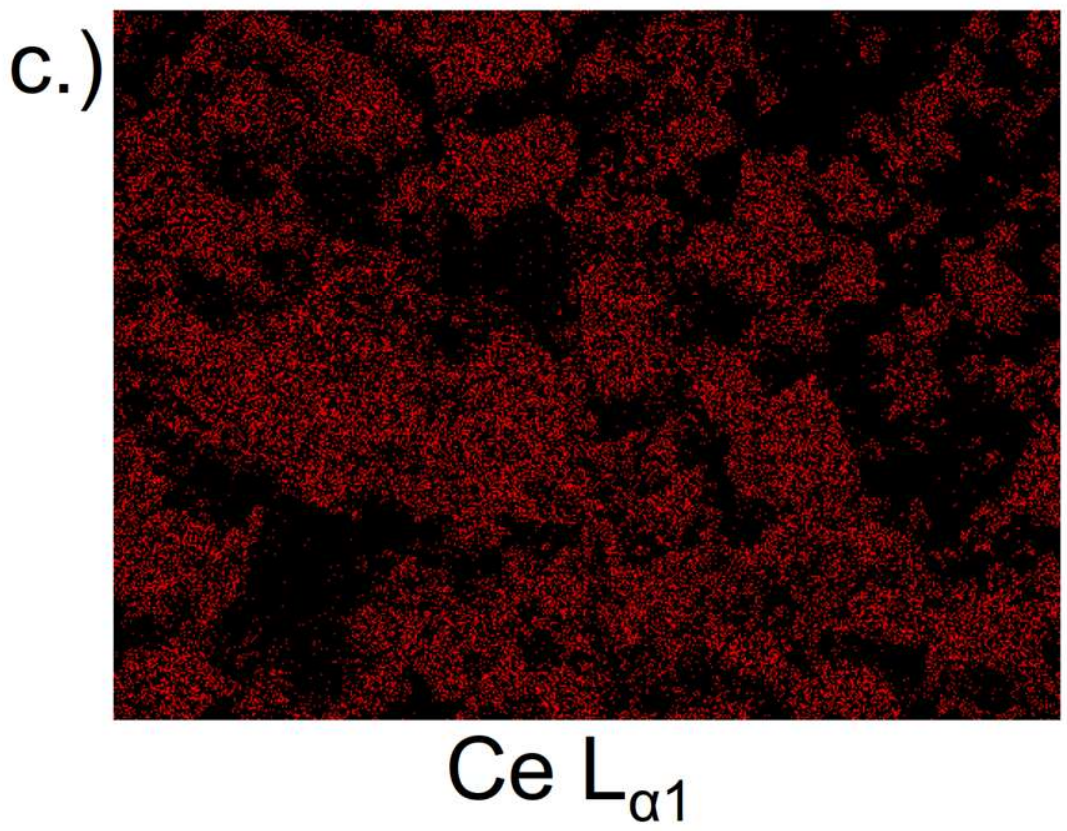

d.)

Number of spectra	8
Atomic-% Ce	55(6) %
Atomic-% Ge	45(6) %

Figure S5. Scanning electron microscope images of CeGe taken at a scale of 1 mm with a quantum back scatter detector (a), the green areas were chosen for EDX analysis and the results averaged (d). EDX mappings for Ge $K_{\alpha1}$ (b) and Ce $L_{\alpha1}$ (c).

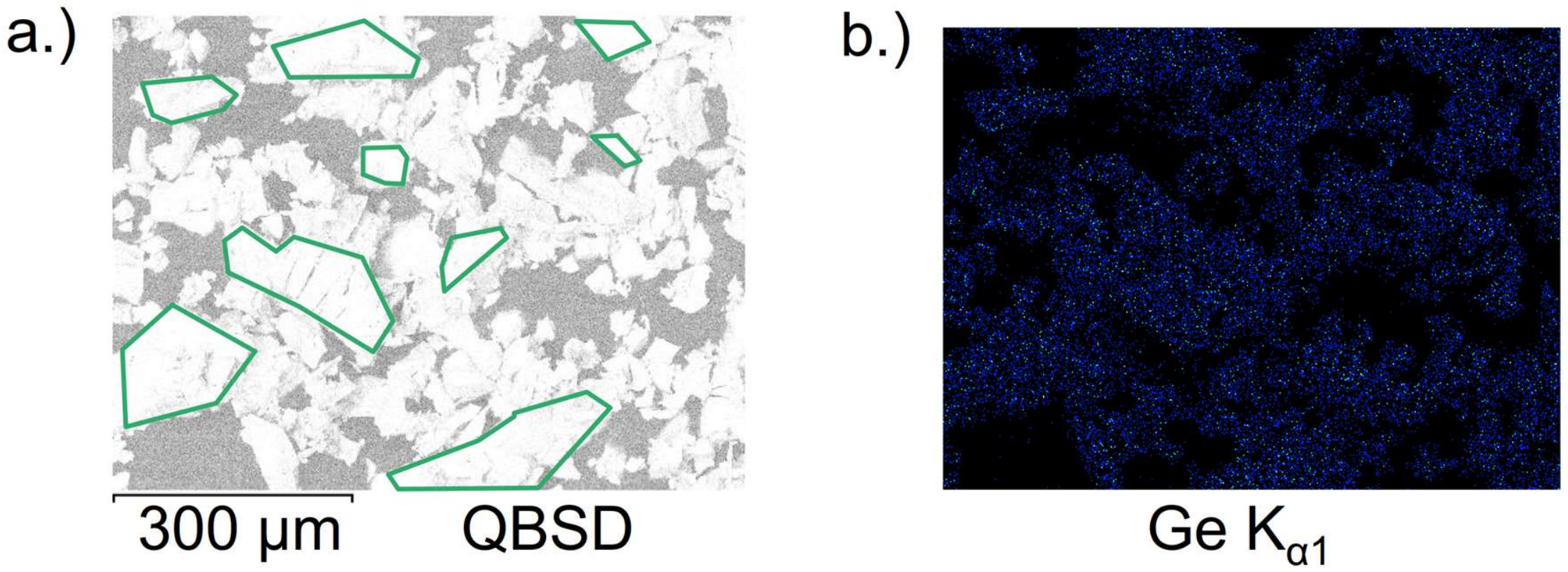

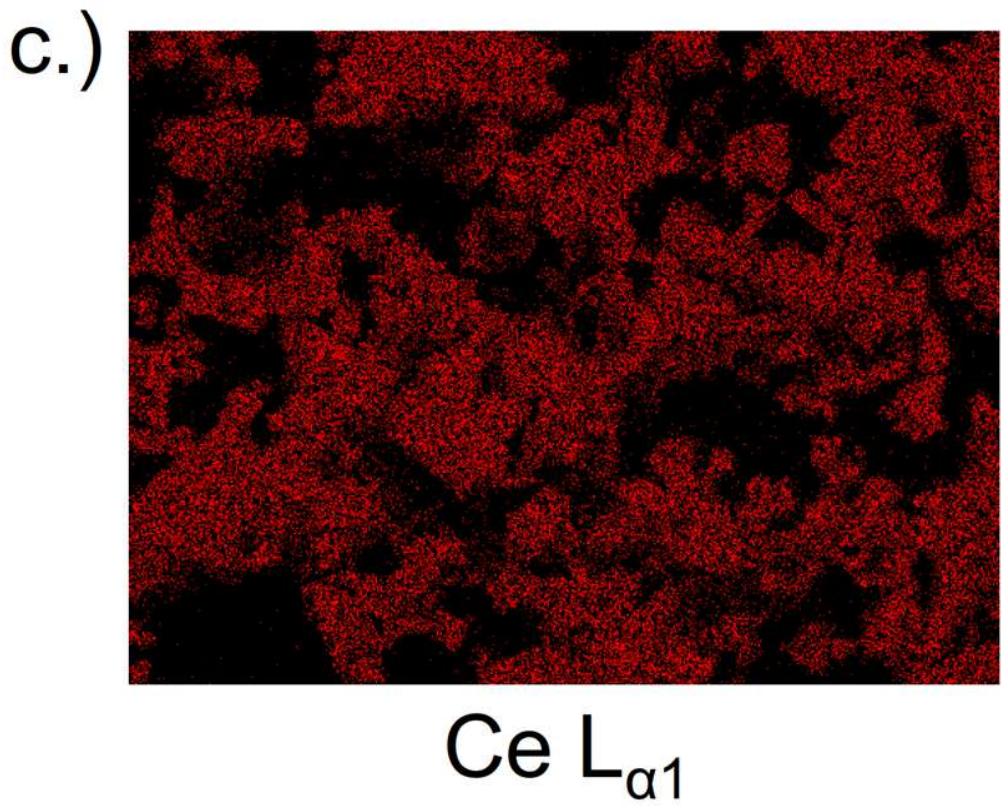

d.)

Number of spectra	9
Atomic-% Ce	55(7) %
Atomic-% Ge	45(7) %

Figure S6. Scanning electron microscope images of $CeGeH_x$ taken at a scale of 300 µm with a quantum back scatter detector (a), the green areas were chosen for EDX analysis and the results averaged (d). EDX mappings for Ge $K_{\alpha1}$ (b) and Ce $L_{\alpha1}$ (c).
